# Overcoming Vaccine Hesitancy for Future COVID-19 and HIV Vaccines: Lessons from Measles and HPV Vaccines

**DOI:** 10.1007/s11904-022-00622-0

**Published:** 2022-09-17

**Authors:** Obianuju G. Aguolu, Amyn A. Malik, Noureen Ahmed, Saad B. Omer

**Affiliations:** 1 Yale Institute for Global Health, Yale University, New Haven, CT USA; 2 Section of Infectious Diseases, Department of Medicine, Yale School of Medicine, Yale University, New Haven, CT USA; 3 Yale School of Public Health, Yale University, New Haven, CT USA

**Keywords:** Human immunodeficiency virus (HIV), Human papillomavirus (HPV), Measles, COVID-19, Interventions, Vaccine hesitancy

## Abstract

**Background:**

The discovery of vaccines significantly reduced morbidity and mortality of infectious diseases and led to the elimination and eradication of some. Development of safe and effective vaccines is a critical step to the control of infectious diseases; however, there is the need to address vaccine hesitancy because of its potential impact on vaccine uptake.

**Methods:**

We conducted a narrative review of studies on interventions to address measles and human papillomavirus vaccine hesitancy. We discussed how lessons learned from these studies could be applied towards COVID-19 and future human immunodeficiency virus vaccines.

**Results:**

We found that there are several successful approaches to improving vaccine acceptance. Interventions should be context specific and build on the challenges highlighted in various settings.

**Conclusion:**

Strategies could be used alone or in combination with others. The most successful interventions directly targeted the population for vaccination. Use of financial incentives could be a potential tool to improve vaccine uptake.

## Introduction

There have been major global efforts to address two highly significant epidemics—human immunodeficiency virus (HIV) and SARS-CoV-2. One of those efforts is the development of vaccines to prevent severe diseases that could result from these infections. Historically, vaccines are the most effective means of preventing illness and death from infectious diseases. The discovery of vaccines has led to reduced morbidity and mortality from several infectious diseases and the elimination and eradication of some [1]. Development of safe and effective vaccines is a huge step to the control of epidemics; however, there is the need to address vaccine hesitancy because of its potential impact on vaccine uptake. Vaccine acceptance is critical in the successful control of pandemics. Successful immunization programs have resulted in the prevention of 4–5 million deaths among all age groups annually, making it one of the most successful and cost-effective public health interventions [2].

WHO defines vaccine hesitancy as a “delay in acceptance or refusal of vaccines despite availability of vaccination services.” The resurgence of infectious diseases in recent years can be attributed to vaccine refusals [3–6]. Vaccine hesitancy are among the 10 threats to global health in 2019 [7–11]. Many individuals are left unvaccinated, leaving them and others around them vulnerable to diseases. With the introduction of the COVID-19 vaccines, there is a greater potential to save millions of lives with vaccines [12]. However, the spread of vaccine misinformation and anti-information has resulted in an increase in vaccine mistrust in the US. False vaccination information is spreading rapidly through social media, causing general distrust and safety concerns, thereby worsening vaccination hesitancy [13] and disease outcomes [14]. There is scientific evidence that anti-vaccination activities organized via social media is predictive of the belief that vaccines are not safe and the prevalence of vaccine disinformation predicts a decrease in mean vaccination coverage over time [15]. Vaccination misinformation may have significant and lasting impacts on vaccine acceptance overall. Poor vaccination rates mean high cases of preventable illnesses, suffering, death [16–22], and huge economic burden [9, 23]. Poor vaccine coverage and the persistent high burden of vaccine-preventable diseases despite proven vaccine safety and effectiveness imply the need to identify drivers of vaccine hesitance and effective intervention strategies to reduce both among this population. Reducing the spread of false vaccine information or the proportion of people who believe them could reduce the effects of these harmful messages [14]. As the world undertakes the largest vaccination campaign in human history to combat COVID-19 and HIV, past research on strategies for vaccine acceptance and hesitancy can inform our understanding of these issues. Lessons learned from interventions to improve measles and human papillomavirus (HPV) vaccine uptake could be applied to improve uptake of COVID-19 vaccine and potential future HIV vaccine(s).

### Human Immunodeficiency Virus (HIV) Vaccine

Since HIV was identified as the cause of acquired immune deficiency syndrome (AIDS) in 1984, millions of people have been affected worldwide. HIV/AIDS incurs huge morbidity, mortality, and economic burden globally. The introduction of the anti-retroviral therapy (ART) resulted in a decrease in the public health and economic burden [24]; however, with a slower decrease in number of new cases, prevalence of the infection continues to rise. Currently, more than 36 million people are infected with HIV and more than 39 million AIDS-related deaths have occurred globally [25]. This is a major public health problem for years to come.

There is currently no vaccine for HIV, but scientists have been working on developing one for over 30 years [26]. The challenges with producing a HIV vaccine lies in the structure and characteristics of the HIV to mutate rapidly and evade the human immune system. This means that a vaccine for one HIV subtype may prevent that specific subtype but be completely ineffective against another HIV subtype. However, extensive progress has been made. The National Institutes for Health (NIH) is investing in a safe and effective preventive HIV vaccine [27]. There is evidence that an upcoming HIV vaccine trial looks promising.

### COVID-19 Vaccine

In December 2019, a cluster of cases of pneumonia was reported in Wuhan, Hubei Province [28]. A novel coronavirus (SARS-CoV-2) was identified as the cause and the disease was called coronavirus disease 2019 (COVID-19). In January 2020, the World Health Organization (WHO) declared COVID-19 a public health emergency of international concern, the highest level of alarm under international law. As of May 2021, more than 160 million confirmed cases of SARS-CoV-2 infections and 3.5 million confirmed deaths had been recorded [29]. In December 2020, the US Food and Drug Administration (FDA) issued the first Emergency Use Authorization (EUA) for the use of a vaccine to protect against COVID-19 and by February 2021, there were three authorized COVID-19 vaccines [30]—Pfizer-BioNTech, Moderna, and Johnson and Johnson vaccines.

Vaccination is a crucial part of ending an epidemic; therefore, widespread vaccine uptake is critical for the control of the global pandemic through vaccine. COVID-19 vaccine has the potential to save lives if its uptake is optimal. Studies show a mixed response to COVID-19 vaccine acceptance globally [31–34] and even among healthcare workers [35, 36]. A study conducted in early 2020 among adults in the United States (US) found a COVID-19 vaccine acceptance rate of 67% [37]. Current data show that as of August 2021, 70% of US adults have received at least one dose of the COVID-19 vaccine [38]; however, there continues to be a geographic and racial/ethnic disparity in the acceptance of the vaccine, with Blacks and Hispanics having persistently low vaccination coverage [39]. In low-income countries, only 1.1% have received at least one dose [40]. Poor COVID-19 vaccine coverage could have negative impacts on the efforts to control the pandemic. There is a need for public health officials and policymakers to prioritize effective strategies to promote acceptance and uptake of COVID-19 vaccines and potential HIV vaccines, especially for those who are most vulnerable. We conducted a narrative review of studies on interventions to address measles and HPV vaccine hesitancy. We discussed how these strategies could be applied towards COVID-19 and potential HIV vaccines.

### Search Strategy

We used a subset of the studies that were retrieved for a different systematic review for this review. We searched the database Medline on the platform Ovid, using the Medline All segment, which includes non-Medline PubMed records. Relevant material from journals not indexed in Medline was identified through citation chaining.

Documents from before 1990 and documents indexed as addressing animals without also addressing humans were not retrieved. When run on March 12, 2020, the search retrieved 14,753 records, of which 47 were later identified as duplicates. The records were uploaded to Covidence for screening. In addition to the database searching, 412 additional potentially relevant studies were identified through citation chaining and uploaded to Covidence for screening. The final search terms used for searching translated to PubMed search query is as follows:

**Table:** 

PubMed translation of final search terms
(vaccines[mh] OR immunization[mh] OR vaccin*[tw] or immunis*[tw] or immuniz*[tw] or inoculat*[tw]) AND (intervention[tw] or interventions[tw] or treatment[tw] or treatments[tw] or group[tw] or groups[tw] or trial[tw] or trials[tw] or program[tw] or programs[tw] or programme[tw] or programmes[tw] or evaluat*[tiab] or experiment*[tiab]) AND (behavior[mh] OR behav*[tw] OR incentiv*[tw] OR psychology[subheading] OR motivate*[tw] OR motivation[mh]) NOT (animals[mh:noexp] NOT humans[mh]) AND (1990:3000[pdat])

### Study Selection

**Fig. 1:**
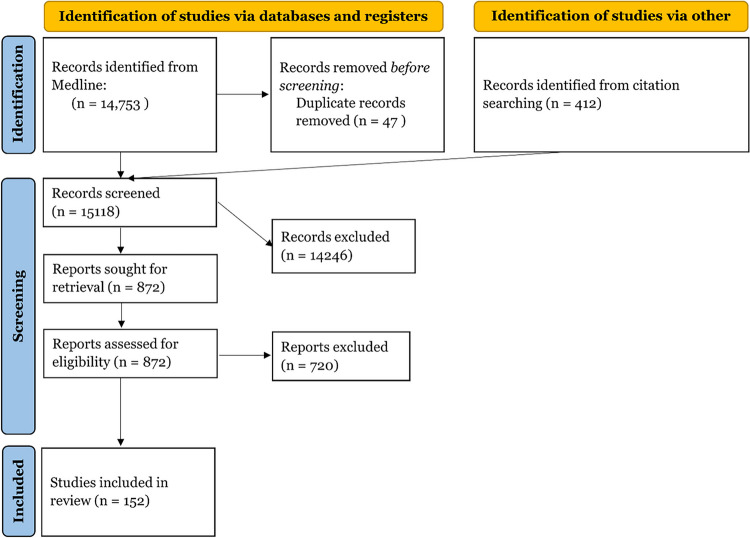
PRISMA flow diagram for HPV and Measles studies

For this review, we included those articles that discussed a behavioral insights intervention on vaccination and excluded those that were explanatory, were not intervention-based, or did not look at vaccine uptake, intent, knowledge, or attitudes as an outcome measure, or were not in English. We also excluded systematic reviews and meta-analyses. We restricted the studies to those that mentioned either measles or HPV in the title or abstract and those that were published after 2005 when the HPV vaccine became available. Two reviewers independently reviewed the records selected for manual screening to identify final studies to include. We included 152 studies on vaccine promotion strategies with diverse contents that were implemented in different settings and targeting various populations (Fig. 1).

### Study Outcomes of Interest

The primary outcome of interest for this review was vaccine uptake with vaccine knowledge, attitudes, and intent as secondary outcomes of interest. Outcome was defined as per each study’s criteria. Some studies classified outcomes based on self-reports while others used clinical or insurance records.

The studies were classified under the 9 types of vaccine promotion intervention domains. These include Education campaigns (providing education on vaccination, disease, and how vaccine work); On-site vaccination (providing vaccine at workplace or places of worship); Incentives (offering financial incentives for vaccination); Free vaccination (providing vaccination free of cost); Institutional recommendation (a recommendation made by the institution that person works at especially for healthcare providers); Provider recommendation (recommendation by doctor/nurse); Reminder and recall (reminders for vaccination); Message framing (gain vs loss framing of the vaccine), and Vaccine champion (institutionally appointed champion that encouraged vaccination). If more than one type of domain was employed, we classified the study as multi-component.

### HPV Vaccine Promotion

We identified 131 articles conducted from 2006 to 2020 that discussed behavioral insights interventions to promote HPV vaccinations (Table 1). Most of the studies were conducted in the US (Table 2). A majority focused on educational interventions, either alone or in combination with one or more strategies. Educational interventions included use of pamphlets, movies, workshops, consulting sessions, and live/virtual sessions to educate on HPV topics and the role of vaccination. The most common target population for the interventions was females, either as HPV vaccine–eligible individuals or their parents/guardians. Some interventions were targeted towards adolescents only [41–53], young adults only [54–60], or a combination of both [61–65], while the rest targeted key decision-makers parents/guardians only [47, 52, 66–74], parent/child dyads [75–79], or parents/healthcare staff/school staff [80]. None of the interventions focused on adolescent boys only. There were also educational interventions that focused on educating healthcare workers [46, 49–51, 62, 81–85]. Some studies used multi-component strategies, i.e., a combination of two or more interventions. Many programs were implemented in schools, universities, or healthcare settings to target adolescents and/or adults.

**Table 1:** Types of HPV vaccine promotion interventions identified in the literature

Type of intervention	No	Authors
Education	60	(Baxter & Barata, 2011), (Chan et al., 2009), (Steckelberg et al., 2013), (Wegwarth et al., 2014), (Kwan et al., 2011), (Marek et al., 2012), (Levinson et al., 2013), (Gottvall et al., 2010), (Lai et al., 2015), (Brabin et al., 2010), (Lloyd et al., 2009), (Shah et al., 2019), (Baldwin et al., 2017), (Bonafide & Vanable, 2015), (Brewer et al., 2017), (Casillas et al., 2011), (Cates et al., 2018), (Chapman et al., 2010), (Cox et al., 2010), (Dempsey et al., 2006), (Diclemente et al., 2011), (Gerend & Barley, 2009), (Groom et al., 2017), (Hopfer, 2012), (Kennedy et al., 2011), (Lee et al., 2018), (McRee et al., 2018), (Obulaney et al., 2016), (Reiter et al., 2011), (Reiter et al., 2018), (Spleen et al., 2012), (Wetzel et al., 2007), (Zimmerman et al., 2017), (Bonville et al., 2019), (Dempsey et al., 2018), (Dempsey et al., 2019), (Dixon et al., 2019), (Gualano et al., 2019), (Hayes et al., 2019), (Kim & Hmielowski, 2017), (Krakow et al., 2017), (Kumar et al., 2019), (Le et al., 2019), (Liu et al., 2019), (Roussos-Ross et al., 2017), (Suarez Mora et al., 2018), (Gilkey et al., 2014), (Choi et al., 2018), (Wadhera et al., 2015), (Chang et al., 2013), (Abuelo et al., 2014), (Bennett et al., 2015), (Chan et al., 2015), (Cipriano et al., 2018), (DiClemente et al., 2015), (Gerend et al., 2013), (Katz et al., 2014), (Perez et al., 2016), (Rickert et al., 2015), (Scherer et al., 2016)
Multi-component	28	(Piedimonte et al., 2018), (Poscia et al., 2019), (Botha et al., 2015), (Meyer et al., 2018), (Reno et al., 2019), (Lefevere et al., 2016), (Fregnani et al., 2013), (Malo et al., 2018), (Gilkey et al., 2016), (Lee et al., 2016), (Berenson et al., 2019), (Tu et al., 2019), (McGlone et al., 2017), (LaMontagne et al., 2011), (Wermers et al., 2021), (Moss et al., 2016), (Zimet et al., 2018), (Grandahl et al., 2016), (Tan et al., 2020), (Fenton et al., 2018), (Sanderson et al., 2017), (Whelan et al., 2014), (Berenson et al., 2016), (Tull et al., 2019), (Goleman et al., 2018), (Perkins et al., 2015), (Deshmukh et al., 2018), (Rand et al., 2018)
Message framing	19	(Juraskova et al., 2011), (Gainforth & Latimer, 2012) (Gainforth et al., 2012), (Leader et al., 2009), (Gesser-Edelsburg et al., 2015), (Nan et al., 2016), (Bell et al., 2014), (Bigman et al., 2010), (Donahue et al., 2018), (Gerend & Shepherd, 2007), (Gerend & Shepherd, 2012), (Gerend et al., 2008), (Juraskova et al., 2012), (Krieger & Sarge, 2013), (Lechuga et al., 2011), (McRee et al., 2010), (Park, 2012)
Reminder and recall	11	(Chao et al., 2015), (Henrikson et al., 2018), (Kempe et al., 2012), (Kempe et al., 2016), (Kreuter et al., 2016), (Mayne et al., 2012), (Patel et al., 2014), (Richman et al., 2016), (Suh et al., 2012), (Szilagyi et al., 2015), (Yee et al., 2017)
Free vaccination program	3	(Widgren et al., 2011), (Harper et al., 2014), (McGrath et al., 2019)
Vaccine champion	3	(Hayashi et al., 2012), (Binagwaho et al., 2012), (Lee et al., 2012)
Financial incentives	2	(Mantzari et al., 2015), (Caskey et al., 2017)
Provider recommendation	2	(Finney Rutten et al., 2017), (Joseph et al., 2016)
On-site vaccination	1	(Golden et al., 2014)
Policy	1	(Dempsey & Schaffer, 2011)
Co-vaccination	1	(Keim-Malpass et al., 2015)
Total	131

**Table 2:** Countries where HPV intervention studies were conducted

Country	No	Authors
US	92	(Keim-Malpass et al., 2015), (Shah et al., 2019), (Baldwin et al., 2017), (Bonafide & Vanable, 2015), (Brewer et al., 2017), (Casillas et al., 2011), (Cates et al., 2018), (Chapman et al., 2010), (Cox et al., 2010), (Dempsey et al., 2006), (Diclemente et al., 2011), (Gerend & Barley, 2009), (Groom et al., 2017), (Hopfer, 2012), (Kennedy et al., 2011), (Lee et al., 2018), (McRee et al., 2018), (Obulaney et al., 2016), (Reiter et al., 2011), (Reiter et al., 2018), (Spleen et al., 2012), (Wetzel et al., 2007), (Zimmerman et al., 2017), (Bonville et al., 2019), (Dempsey et al., 2018), (Dempsey et al., 2019), (Dixon et al., 2019), (Gualano et al., 2019), (Hayes et al., 2019), (Kim & Hmielowski, 2017), (Krakow et al., 2017), (Kumar et al., 2019), (Roussos-Ross et al., 2017), (Suarez Mora et al., 2018), (Gilkey et al., 2014), (Choi et al., 2018), (Bennett et al., 2015), (Chan et al., 2015), (Cipriano et al., 2018), (DiClemente et al., 2015), (Gerend et al., 2013), (Katz et al., 2014), (Perez et al., 2016), (Rickert et al., 2015), (Scherer et al., 2016), (Caskey et al., 2017), (Harper et al., 2014), (Nan et al., 2016), (Bell et al., 2014), (Bigman et al., 2010), (Donahue et al., 2018), (Gerend & Shepherd, 2007), (Gerend & Shepherd, 2012), (Gerend et al., 2008), (Juraskova et al., 2012), (Krieger & Sarge, 2013), (Lechuga et al., 2011), (McRee et al., 2010), (Park, 2012), (Malo et al., 2018), (Reno et al., 2019), (Gilkey et al., 2016), (Berenson et al., 2019), (Meyer et al., 2018), (McGlone et al., 2017), (Sanderson et al., 2017), (Wermers et al., 2021), (Zimet et al., 2018), (Tan et al., 2020), (Fenton et al., 2018), (Moss et al., 2016), (Berenson et al., 2016), (Goleman et al., 2018), (Perkins et al., 2015), (Deshmukh et al., 2018), (Lee et al., 2016), (Rand et al., 2018), (Golden et al., 2014), (Dempsey & Schaffer, 2011), (Finney Rutten et al., 2017), (Joseph et al., 2016), (Chao et al., 2015), (Henrikson et al., 2018), (Kempe et al., 2012), (Kempe et al., 2016), (Kreuter et al., 2016), (Mayne et al., 2012), (Patel et al., 2014), (Richman et al., 2016), (Suh et al., 2012), (Szilagyi et al., 2015), (Yee et al., 2017)
Canada	5	(Baxter & Barata, 2011), (Gainforth & Latimer, 2012), (Gainforth et al., 2012), (Piedimonte et al., 2018), (Whelan et al., 2014)
China	4	(Chan et al., 2009), (Liu et al., 2019), (Chang et al., 2013), (Liu et al., 2019)
Australia	3	(McGrath et al., 2019), (Juraskova et al., 2011), (Tull et al., 2019)
Taiwan	3	(Lai et al., 2015), (Tu et al., 2019), (Lee et al., 2012)
UK	3	(Mantzari et al., 2015), (Brabin et al., 2010), (Lloyd et al., 2009)
Peru	2	(Levinson et al., 2013), (Abuelo et al., 2014)
Sweden	2	(Gottvall et al., 2010), (Grandahl et al., 2016)
Germany	2	(Steckelberg et al., 2013), (Wegwarth et al., 2014)
Hong Kong	2	(Kwan et al., 2011), (Leader et al., 2009)
Vietnam	2	(Le et al., 2019), (Lee & Cho, 2017)
Cambodia	1	(Wadhera et al., 2015)
Rwanda	1	(Binagwaho et al., 2012)
Brazil	1	(Fregnani et al., 2013)
Israel	1	(Gesser-Edelsburg et al., 2015)
South Africa	1	(Botha et al., 2015)
Italy	1	(Poscia et al., 2019)
Belgium	1	(Lefevere et al., 2016)
Japan	1	(Hayashi et al., 2012)
Denmark	1	(Widgren et al., 2011)
Hungary	1	(Marek et al., 2012)
India, Peru, Uganda, Vietnam	1	(LaMontagne et al., 2011)
Total	**131**	

Some authors focused on improving HPV vaccine uptake for specific populations such as girls only [43, 44, 47, 72, 75, 76, 78, 80, 86–89], young men only [55, 56, 90], or young women only [54, 91–97], college students [55, 58, 90–92, 97, 98], gay and bisexual men [80, 99, 100], female entertainment sex workers [101], and ethnicity/races [59, 60, 62, 76, 77, 89] (Table 3). Fewer studies targeted specific groups such as a race/ethnicity or a gender. The common outcome variables studied include intention to get HPV vaccine, actual HPV vaccine uptake, HPV or HPV vaccine awareness or knowledge, risk perception for HPV, attitudes towards HPV vaccination, perceived norm, and self-efficacy to get vaccinated against HPV.

**Table 3:** Populations targeted for HPV vaccination

Population	No	Authors
Adolescents	52	(Cates et al., 2018), (Chang et al., 2013) (Liu et al., 2019), (Shah et al., 2019), (Baldwin et al., 2017), (Brewer et al., 2017), (Chapman et al., 2010), (Dempsey et al., 2006), (Groom et al., 2017), (Zimmerman et al., 2017), (Bonville et al., 2019), (Dempsey et al., 2018), (Dixon et al., 2019), (Kumar et al., 2019), (Gilkey et al., 2014), (Cipriano et al., 2018), (Scherer et al., 2016), (Marek et al., 2012), (Choi et al., 2018), (Rickert et al., 2015), (Gottvall et al., 2010), (Lloyd et al., 2009), (Cox et al., 2010), (Wetzel et al., 2007), (Katz et al., 2014), (Caskey et al., 2017), (Gainforth et al., 2012), (Nan et al., 2016) (Bigman et al., 2010), (Liu et al., 2019), (Poscia et al., 2019), (Malo et al., 2018), (Reno et al., 2019), (Gilkey et al., 2016), (Berenson et al., 2019), (Tu et al., 2019), (Zimet et al., 2018), (Grandahl et al., 2016), (Fenton et al., 2018), (Whelan et al., 2014), (Tull et al., 2019), (Goleman et al., 2018), (Lee et al., 2016), (Rand et al., 2018), (Golden et al., 2014), (Finney Rutten et al., 2017), (Kempe et al., 2016), (Henrikson et al., 2018), (Kempe et al., 2012), (Szilagyi et al., 2015), (Donahue et al., 2018), (Lechuga et al., 2011)
Adolescents (girls only)	28	(Lai et al., 2015), (Steckelberg et al., 2013), (Kwan et al., 2011), (Wegwarth et al., 2014), (Levinson et al., 2013) (Abuelo et al., 2014), (Reiter et al., 2011), (Spleen et al., 2012), (Chan et al., 2009), (Kennedy et al., 2011), (Mantzari et al., 2015), (Widgren et al., 2011), (Gesser-Edelsburg et al., 2015), (Lefevere et al., 2016), (LaMontagne et al., 2011), (Fregnani et al., 2013), (Moss et al., 2016), (Dempsey & Schaffer, 2011), (Joseph et al., 2016), (Mayne et al., 2012), (Suh et al., 2012), (Hayashi et al., 2012), (Brabin et al., 2010), (Casillas et al., 2011), (Botha et al., 2015), (McGlone et al., 2017), (Binagwaho et al., 2012), (Lee et al., 2012)
Adults	9	(Gualano et al., 2019), (Hayes et al., 2019), (Gerend & Shepherd, 2007), (Lee & Cho, 2017), (Piedimonte et al., 2018), (Wermers et al., 2021), (Tan et al., 2020) (Kreuter et al., 2016), (Park, 2012)
Adults (women only)	9	(Kim & Hmielowski, 2017), (Krakow et al., 2017), (Le et al., 2019), (Juraskova et al., 2011), (Gainforth & Latimer, 2012), (Gerend et al., 2008), (Yee et al., 2017), (Patel et al., 2014), (Gerend et al., 2013)
Adults (university women only)	6	(Gerend & Shepherd, 2012), (Bennett et al., 2015), (Perez et al., 2016), (Juraskova et al., 2012), (Krieger & Sarge, 2013), (Hopfer, 2012)
Adults and adolescents	6	(Leader et al., 2009), (Perkins et al., 2015), (Deshmukh et al., 2018), (Keim-Malpass et al., 2015), (Roussos-Ross et al., 2017), (Meyer et al., 2018)
Adults and adolescents (females only)	4	(Suarez Mora et al., 2018), (Harper et al., 2014), (Berenson et al., 2016), (Chao et al., 2015)
Adults (university men only)	3	(Gerend & Barley, 2009), (Bonafide & Vanable, 2015), (Diclemente et al., 2011)
Adults (gay and bisexual men only)	3	(McRee et al., 2018), (McGrath et al., 2019), (Reiter et al., 2018)
Adults (university students only)	2	(Bell et al., 2014), (Richman et al., 2016)
Adults (men only)	1	(McRee et al., 2010)
Adults (sexually inexperienced women only)	1	(Baxter & Barata, 2011)
Adults (Hispanic only)	1	(Chan et al., 2015)
Adults and adolescents (Latino only)	1	(Dempsey et al., 2019)
Adolescents (African American and Hispanic only)	1	(Sanderson et al., 2017)
Adolescents (girls) (Cambodian American only)	1	(Lee et al., 2018)
Adolescents (girls) (Hispanic only)	1	(Obulaney et al., 2016)
Adults (young female entertainment and sex workers)	1	(Wadhera et al., 2015)
Adolescents (girls) (African American only)	1	(DiClemente et al., 2015)
Adults (Hispanic only)	1	(Chan et al., 2015)
Total	**131**	

Overall, educational interventions improved HPV or HPV vaccine awareness and knowledge [41–44, 66, 67, 75, 86–88], beliefs [41], risk perception for HPV [87], perceived effectiveness of HPV vaccine [69], attitudes towards HPV vaccination [41–43, 45, 67, 75, 86, 88], self-efficacy to get vaccinated against HPV, perceived norms [88], acceptance or intention to get vaccinated against HPV [43–45, 54, 55, 66, 67, 75, 88], and actual HPV vaccine uptake [46, 68, 75, 81, 82]. Improved awareness and knowledge of HPV and HPV vaccine were usually associated with intention to get vaccinated or actual vaccine uptake; however, some studies found non-significant difference in HPV vaccine acceptability after an educational intervention [71]. Educational interventions designed to target specific populations were effective in improving vaccine knowledge and this was usually associated with improved vaccine acceptance. Parents and caregivers were more confident in HPV vaccine when they were exposed to messages that addressed lack of knowledge about the vaccine [52, 67, 72, 74, 75, 77–79, 83]. Some of the educational interventions targeted providers, while others targeted young adults as catch-up populations.

The second most common intervention was message framing [64, 102–119]. This involves describing the benefits of receiving (gain-framed message) or the costs of not receiving (loss-framed message) HPV vaccine to the participants. This type of intervention was employed mainly for adults. The effect of message framing on HPV vaccine acceptance was moderated by perceived susceptibility [106, 110, 112] in some cases. Overall, message framing improved HPV knowledge and HPV vaccine acceptance; however, this depended on the other variables in the study and use of appropriately framed messages for target populations. Some authors found that HPV vaccination intention and uptake were not influenced by message framing [102, 111]. Furthermore, some authors found some types of message framing to have negative effects on vaccine acceptance [103]. Gainforth and Latimer found that participants (women) who were exposed to high-risk information perceived HPV vaccine as having higher response cost and were less motivated to receive HPV vaccine compared to women who received low-risk information. The authors observed that this negative effect of high-risk information may be mitigated by appropriately framed messages. Increase in vaccination intentions and uptake significantly improved when messages were tailored to the target population [90].

The use of financial incentives improved HPV vaccine initiation and completion in England [120] and the US [121]. Impacts were not moderated by deprivation level and decision quality was unaffected by the intervention. Offering financial incentives to the participants was associated with significant increase in both the initiation and completion of HPV vaccines series.

Free-of-charge HPV vaccine was associated with increased vaccine uptake in some settings. In a study conducted in Australia, when HPV vaccine was offered free of charge, there was a significant increase in vaccine coverage from 42.6 to 73.2% among gay and bisexual men [122]. In Denmark, free vaccination increased coverage for adolescent girls to 80% for dose 1, 75% for dose 2, and 62% for dose 3 [122]. High-risk participants or those who perceived themselves to be at risk were more likely to accept the free vaccine [100]. On the other hand, the study in the US found that the proportion of adolescent and adult females completing three on-time HPV4 doses was 21% vs. 18% respectively. No adolescent receiving free HPV4 vaccine completed three doses. Grant sponsorship of at least one HPV4 dose among adults did not predict three dose on-time completion (OR = 1.56, 95%CI: 0.80, 3.06). Neither was adult grant sponsorship of HPV4 significant when analyzing exclusive payor sources vs. a combination of payor sources (OR = 0.72, 95%CI: 0.10, 5.17).

Most of the studies that employed multi-component strategies included education of target populations in addition to one or more other domains, e.g., on-site vaccination [123–127], vaccine champion [124, 128, 129], free vaccination [124, 125], reminder and recall [129–134], incentives [135, 136], provider recommendation [126, 130, 137, 138], and provider/staff training sessions [138]. Some studies combined message framing with one or more strategies such as free vaccine [139], and reminder and recall [140]. Another popular intervention was provider recommendation, either alone [141] or in combination with one or more strategies such as provider education [142, 143], reminder and recall [144–147], on site vaccination [148–150], incentive [146], vaccine champion [147], and free vaccination [147]. Tull et al. combined reminder and recall strategy with vaccination champion and on-site vaccination [151]. Multi-component strategies were used in variety of settings including school-based [124, 125, 127, 128, 133, 139, 142, 148, 151], healthcare [129, 130, 132, 134, 136, 137, 145–147, 150], and public settings [126, 131, 135, 144]. Target populations for multi-component strategies include adults as either vaccine decision-makers for adolescents or catch-up populations [123, 142], adolescents [123, 126, 128, 130–132, 134, 137, 138, 145, 146, 148, 150, 151], adolescent girls only [125, 127, 135, 140, 144], both adults and adolescents [129, 136, 147, 152], and healthcare providers [137]. Multi-component strategies were successful in improving HPV knowledge among adults and adolescents [131, 139], healthcare providers’ attitudes, subjective norms about HPV vaccination, and their perceived behavioral control to recommend HPV vaccination [137], as well as vaccine acceptance among parents/guardians [126, 127, 130, 131, 140, 144, 150] and adolescents [126, 131, 139, 148]. It significantly improved vaccine initiation and completion among girls [124, 125, 127, 135, 144], young adults [123, 142], adolescents [123, 126, 128, 131–134, 138, 145, 146, 150, 151], and adults and adolescents [126, 129, 136, 147, 152]. Our findings indicate that multi-component strategies were efficient for all study settings and target populations, and significantly had positive effects on outcome variables.

The use of patient reminder system alone was acceptable to parents [153, 154] and increased rates of vaccine initiation and completion among adolescent girls [155, 156], adolescents [153, 156–159], young adult females [156, 160–162], and young adults [163]. It increased vaccine intention in adults [154, 163]. However, some authors found no significant increase in completion rates [160] and no significant difference in the completion rates between intervention and control groups [153, 158, 160, 161]. The effect of this strategy appeared to be stronger in girls aged 9–17 years compared with young women aged 18–26 years at the first dose and in blacks compared with whites [156].

Use of vaccine champions were found to be successful in improving HPV vaccine coverage up to the 80 s or 90 s percentage among adolescent girls [125, 127, 164–166].

### Measles Vaccine Promotion

We identified 22 studies focused on measles vaccine promotion interventions of which 6 focused on education only, 6 on message framing, 2 on health systems only, 2 on financial incentives only, 1 on reminder and recall only, 1 on on-site/closer vaccination only, and 1 on provider recommendation only. Three of the studies employed multi-component strategies (Table 4). Studies focused on educational interventions found that use of a decision aid [167], using spiritual leaders [168], and combining education with on-site vaccination [169] (campaigns) can increase measles vaccination coverage. Studies on messaging found that both gain-framed and loss-framed messaging and messages focused on disease risk can improve intent to vaccinate their children in parents [170–173]. Financial incentives both at the level of the provider and at the level of the consumer can improve vaccine uptake [174–177]. There is also some evidence suggesting that there might be a dose response relationship between vaccine uptake and monetary amount for incentives being offered to the consumer [177]. Reminder and recall [178] strategy alone did not improve vaccine uptake but when paired with financial incentives [175, 177], significantly improved vaccination rates. Studies that focused on improving health system performance and quality also led to improved vaccination coverage [179, 180]. Distance to healthcare facilities is a predictor of service usage including vaccination coverage and can also exacerbate inequities. One study [181] focused on optimal location of vaccination service delivery found that after placing outreach points for vaccination, there was no relationship between distance to health facility and vaccination coverage for measles highlighting the importance of on-site/closer vaccination points (Tables 5 and 6).

**Table 4:** Types of measles vaccine promotion interventions identified from the literature

Domain	No	Authors
Education	6	(Wallace, 2006), (Majdzadeh, 2008), (Shourie, 2013), (Abdul Rahman, 2013), (Lu, 2017), (Moss, 2012)
Message framing	6	(Abhyankar, 2008), (Nyhan, 2014), (Hendrix, 2014), (Horne, 2015), (Reavis, 2017), (Powell-Jackson, 2018)
Health Systems	2	(Fu, 2012), (Goel, 2012)
Multi-component (reminder and recall; incentives)	2	(Cockman, 2011)
Incentives	2	(Barham, 2009), (Merilind, 2015)
Reminder and recall	1	(Hofstetter, 2015)
Provider recommendation	1	(Ruiz-Cuesta, 2016)
On-site vaccination	1	(Sasaki, 2011)
Multi-component (education; onsite vaccination)	1	(Uddin, 2016)
Total	**22**

**Table 5:** Countries where measles intervention studies were conducted

Country	No	Authors
US	4	(Moss, 2012), (Reavis, 2017), (Fu, 2012), (Nyhan, 2014), (Hendrix, 2014), (Horne, 2015), Hofstetter, 2015)
UK	3	(Shourie, 2013), (Cockman, 2011), (Abhyankar, 2008)
India	2	(Goel, 2012), (Powell-Jackson, 2018)
Australia and New Zealand	1	(Wallace, 2006)
Spain	1	(Ruiz-Cuesta, 2016)
Nicaragua	1	(Barham, 2009)
China	1	(Lu, 2017)
Iran	1	(Majdzadeh, 2008)
Estonia	1	(Merilind, 2015)
Zambia	1	(Sasaki, 2011)
Iraq	1	(Abdul Rahman, 2013)
Bangladesh	1	(Uddin, 2016)
Kenya	1	(Gibson, 2017)
Total	**22**

**Table 6:** Population targeted for measles or MMR vaccination

Population	No	Authors
Children	19	(Uddin, 2016), (Lu, 2017), (Merilind, 2015), (Goel, 2012), (Powell-Jackson, 2018), (Abdul Rahman, 2013), Gibson, 2017), (Barham, 2009 Shourie, 2013), (Cockman, 2011), (Abhyankar, 2008), (Reavis, 2017), (Fu, 2012), (Horne, 2015), (Hofstetter, 2015), (Sasaki, 2011), (Wallace, 2006), (Nyhan, 2014), (Hendrix, 2014)
Children and adults	1	(Majdzadeh, 2008)
Adults	1	(Ruiz-Cuesta, 2016)
Adolescents	1	(Moss, 2012)
Grand Total	**22**	

## Conclusion

### Lessons for COVID-19 and Potential HIV Vaccines

Review of the studies on improving measles and HPV vaccinations shows that the most promising strategies include use of multi-component interventions especially when education is included. Educational interventions focused on disease risk can be used to improve coverage of COVID-19 and a future HIV vaccine in diverse populations. Message framing is a powerful tool for vaccine promotion; however, messages should be carefully framed and should be targeted to the population of interest. Financial incentives, free-of-charge vaccines, and use of vaccine champions should be considered in future vaccine promotions as they were successful in increasing both measles and HPV vaccine coverage rates. Interventions should be context specific, be designed to target populations, and build on the challenges highlighted in various settings.

## Abbreviations

HPV: Human papillomavirus
HIV: Human immunodeficiency virus
AIDS: Acquired immune deficiency syndrome
COVID-19: Coronavirus disease-2019
SARS-CoV-2: Severe acute respiratory syndrome coronavirus-2
MMR: Measles, mumps, and rubella
